# Insomnia and Benzodiazepine Use as Risk Factors for Erectile Dysfunction: Clinical Evidence and In Silico Analysis of Physicochemical Properties

**DOI:** 10.3390/jcm14196951

**Published:** 2025-10-01

**Authors:** Valeria Navarrete-Anaya, Iván Delgado-Enciso, Gustavo A. Hernández-Fuentes, Janet Diaz-Martinez, Osiris G. Delgado-Enciso, Ana Sánchez-Arizmendi, Alejandro Figueroa-Gutiérrez, José Aguilar-Cota, Jesús Venegas-Ramírez, Patricia Calvo-Soto, Karla B. Carrazco-Peña, Mercedes Fuentes-Murguia, Mónica Ríos-Silva, José Guzmán-Esquivel

**Affiliations:** 1 Departamento de Geriatría, Instituto Mexicano del Seguro Social (IMSS), Hospital de Zona No. 1, Villa de Alvarez 28984, Mexico; navarretevaleria32@gmail.com (V.N.-A.); analysa339@gmail.com (A.S.-A.); dr.aguilarcota@gmail.com (J.A.-C.); 2 Department of Molecular Medicine, School of Medicine, University of Colima, Colima 28040, Mexico; ivan_delgado_enciso@ucol.mx (I.D.-E.); gahfuentes@gmail.com (G.A.H.-F.); 1933osiris@gmail.com (O.G.D.-E.); dra_carrazco@ucol.mx (K.B.C.-P.); fuentes_murguia@ucol.mx (M.F.-M.); mrios@ucol.mx (M.R.-S.); 3 State Cancerology Institute of Colima, Health Services of the Mexican Social Security Institute for Welfare (IMSS-BIENESTAR), Colima 28085, Mexico; calvosotopatricia@gmail.com; 4 Department of Dietetics & Nutrition, Robert Stempel College of Public Health and Social Work, Florida International University, Miami, FL 33199, USA; jdimarti@fiu.edu; 5 Faculty of Chemical Sciences, University of Colima, Coquimatlan 28400, Mexico; 6 Research Center in Minority Institutions, Florida International University (FIU-RCMI), Miami, FL 33199, USA; 7 Health Education Auxiliary Coordination, Mexican Institute of Social Security (IMSS), Villa de Alvarez 28984, Mexico; alejandro.figueroag@imss.gob.mx; 8 Department of Nephrology, Instituto Mexicano del Seguro Social (IMSS), Hospital de Zona No. 1, Villa de Alvarez 28984, Mexico; nefrojesusvr@gmail.com; 9 Clinical Epidemiology Research Unit, Mexican Institute of Social Security, Villa de Alvarez 29883, Mexico

**Keywords:** erectile dysfunction, insomnia, benzodiazepine use, risk factors, cross-sectional study, physicochemical descriptors, structure-relation analysis

## Abstract

**Background/Objectives:** Erectile dysfunction (ED) is a prevalent and multifactorial condition influenced by psychological and sleep-related factors. This study aimed to evaluate the independent and combined associations of insomnia and benzodiazepine use with the risk of ED. **Methods:** An analytical cross-sectional study was conducted in adult men with and without ED. Logistic regression was used to estimate crude and adjusted odds ratios (ORs). Effect modification was assessed through stratified analyses. Additionally, an in silico analysis of 17 active compounds was performed using SwissADME and Molinspiration to explore physicochemical properties. **Results:** Insomnia (adjusted OR 2.05; 95% CI 1.13–3.74; *p* = 0.019) and benzodiazepine use (adjusted OR 2.14; 95% CI 1.10–4.15; *p* = 0.025) were each independently associated with ED. In contrast, antidepressant use was not significantly associated with ED in the sample analyzed. Participants with both insomnia and benzodiazepine use had a markedly higher risk (adjusted OR 3.96; 95% CI 1.51–10.40; *p* = 0.005). The joint association of insomnia and benzodiazepine use was consistent with the combined effect expected from their individual associations. The in silico analysis showed an overlapping profile, suggesting benzodiazepine properties may underline their link to ED, supporting the results of the cross-sectional study. **Conclusions:** Both insomnia and benzodiazepine use independently increased the odds of ED. Their co-occurrence was linked to a substantially higher likelihood of ED, highlighting the clinical importance of assessing both conditions concurrently in patients with sexual dysfunction.

## 1. Introduction

Erectile dysfunction (ED), defined as the persistent inability to attain or maintain penile erection sufficient for satisfactory sexual intercourse [1], represents a highly prevalent condition globally. Large population-based studies, such as the Massachusetts Male Aging Study (MMAS) and the European Male Aging Study (EMAS), have reported a prevalence of 52% in men aged 40–70 years [2], and overall global prevalence is substantial [3]. ED is known to be multifactorial, associated with numerous medical conditions, including cardiovascular diseases, diabetes, obesity, and hypertension, as well as psychological factors [1].

Insomnia, characterized by difficulty initiating or maintaining sleep or experiencing non-restorative sleep [4], often with daytime impairment lasting at least three months despite adequate opportunity for sleep [4], is a common sleep disorder affecting a significant proportion of the adult population. Its prevalence varies across studies and diagnostic criteria, but reviews indicate substantial rates [5]. For instance, prevalence estimates for insomnia symptoms or clinical insomnia range widely depending on the definition and population studied [6,7].

Emerging evidence suggests a strong association between sleep disorders—particularly insomnia—and male sexual function [7]. A recent large-scale analysis of insurance claims data from 2007 to 2016, involving over 500,000 men diagnosed with insomnia, investigated this relationship [4]. The prevalence of difficulty initiating or maintaining sleep increases linearly with age, affecting nearly 50% of individuals over 65 years old [8]. The study reported that men diagnosed with insomnia had a 1.58 times greater likelihood of receiving a diagnosis of erectile dysfunction (ED) compared to age-matched controls without insomnia (Hazard Ratio [HR] 1.58; 95% CI 1.54–1.62; *p* < 0.001) [4]. Furthermore, men who were both diagnosed and treated for insomnia had an even higher risk, with a 1.66 times greater likelihood of ED diagnosis (HR 1.66; 95% CI 1.64–1.69; *p* < 0.001). Treatment for insomnia was also associated with an increased likelihood of ED being treated with phosphodiesterase-5 inhibitors (HR 1.52) and intracavernosal injections (HR 1.32) [4]. Other studies using Mendelian randomization have identified insomnia as a highly relevant risk factor for ED (OR = 3.44; 95% CI = 1.59–7.43) [9]. These findings underscore the importance of considering insomnia during the clinical evaluation of patients presenting with erectile dysfunction.

Concurrently, pharmacological treatments for psychiatric conditions, particularly antidepressants and benzodiazepines, are well established causes of treatment-emergent sexual dysfunction (TESD) [2,6,10,11,12]. Selective serotonin reuptake inhibitors (SSRIs) and serotonin–norepinephrine reuptake inhibitors (SNRIs) are among the most frequently implicated antidepressant classes, associated with decreased libido, delayed ejaculation, and erectile difficulties [2,6,10]. TESD is a common adverse effect that can compromise treatment adherence and quality of life [2,6,10,11].

Benzodiazepines, widely prescribed for anxiety and insomnia, have also been associated with sexual dysfunction, including erectile dysfunction (ED). Their central nervous system depressant effects may interfere with arousal pathways, sexual desire, and performance [12,13]. Furthermore, benzodiazepine use frequently coexists with sleep disorders and mood disturbances, making it difficult to disentangle the contribution of underlying psychiatric conditions from medication-related effects.

The complex interplay between mental health pharmacological treatment, insomnia, and sexual function is increasingly recognized. Pharmacological treatment, insomnia, and sexual function is increasingly recognized [10,12]. Men receiving pharmacological therapy for insomnia were more likely to be prescribed medications for erectile dysfunction [4]. Considering how common insomnia is, further research is needed to clarify how both insomnia and its treatment may impact erectile function. Understanding these multifactorial associations is essential to guide clinical decision-making in populations at high risk of sexual health impairments. Our objective was to analyze the independent associations and possible variation in the strength of the relationship (effect modification) between insomnia and benzodiazepine use with the risk of erectile dysfunction across different subgroups.

## 2. Materials and Methods

### 2.1. Study Design and Participants

An analytical cross-sectional observational study was conducted in which adult men over 65 years of age, not hospitalized, participated. Participants were consecutively recruited from general medical outpatient consultations from General Hospital of Zone 1 (Villa de Alvarez, Colima, México) between January-December 2024. All participants provided written informed consent prior to participation. The study protocol was approved by the institutional ethics committee (Registration number: R-2024-601-001, 26 January 2024). Inclusion criteria included age ≥65 years and availability of complete data on sexual function, comorbidities, and mental health. Exclusion criteria were severe cognitive impairment, active psychiatric disorders requiring hospitalization, and current malignant disease. All information collected for this study was obtained through direct interviews with participants and information obtained from the clinical record. The study followed the guidelines for reporting observational studies (STROBE) [14,15].

### 2.2. Assessment of Erectile Dysfunction

Erectile dysfunction (ED) was assessed using the International Index of Erectile Function-5 (IIEF-5), a validated instrument for evaluating male erectile function. Participants were categorized as having or not having ED based on the scoring algorithm defined by the IIEF-5, with lower scores reflecting greater sexual dysfunction. For the purposes of this analysis, ED was treated as a dichotomous variable based on established cut-off points (≤21) [16].

### 2.3. Clinical and Psychosocial Variables

Sociodemographic and clinical variables were obtained through structured interviews and medical record review. These included age (analyzed both as a continuous variable and dichotomized as ≥75 years), presence of type 2 diabetes mellitus (DM2), systemic arterial hypertension (HTN), current alcohol consumption, lifetime tobacco use, insomnia, depression, antidepressant use, and benzodiazepine use. Tobacco use was defined according to the World Health Organization (WHO) criteria as a lifetime history of smoking 100 or more cigarettes. Alcohol use was coded as current consumption (yes/no). Depression was defined as having 5 or more points on the Geriatric Depression Scale (GDS) administered at the time of all participant assessments. If the patient was taking antidepressants but did not comply with the above criteria, they were classified as not depressed. The GDS is a screening tool for assessing depression in older adults [17]. Antidepressant or benzodiazepine use were recorded based on current pharmacologic treatment at the time of interview. Insomnia was assessed using the Athens Insomnia Scale (AIS), a validated tool for measuring sleep disturbances [18]. The AIS score is useful for detecting the presence and severity of insomnia. The AIS consists of 8 items that assess sleep difficulties and daytime consequences, with scores ranging from 0 to 24. A score of ≥6 suggests clinically significant insomnia [18], and the total score reflects the severity of the condition [18,19].

### 2.4. Sample Size and Power Calculation

The sample size was estimated using the formula for the unpaired cross-sectional study design, based on the risk of insomnia for developing ED found in a previous study (OR 3.44) [9], considering a hypothetical proportion of controls with insomnia of 50%, as reported in previous studies [8]. Using a significance level (α) of 0.05 and a statistical power (1-β) of 80%, the required sample size for comparing the groups was calculated to be 49 patients per group (with and without ED). Upon completion of this study, a post hoc statistical power analysis was conducted. The analysis revealed that having insomnia and taking benzodiazepine (both factors) significantly increase the likelihood of ED. The statistical power for this research was 83.8%.

### 2.5. Structure–Activity Relationship Analysis

A physicochemical descriptor analysis was conducted to compare the molecular profiles of 17 pharmacologically active compounds with relevance to sexual function modulation. These included the following: Benzodiazepines (clonazepam, diazepam, lorazepam, midazolam, alprazolam, chlordiazepoxide, oxazepam, temazepam, flurazepam, and triazolam). Selective serotonin reuptake inhibitors (SSRIs) (fluoxetine, paroxetine, escitalopram, and fluvoxamine). Beta-blockers (propranolol). Phosphodiesterase type 5 (PDE5) inhibitors: (sildenafil and tadalafil). A total of 25 molecular descriptors were calculated using the SwissADME online platform and Molinspiration tools, focusing particularly on those relevant to polarity, lipophilicity, and membrane permeability [20]. These included topological polar surface area (TPSA), predicted skin permeability (log Kp), and consensus log P, which were used to identify contrasting pharmacological profiles related to sexual function modulation. The selection was intentional and justified to include both: drugs frequently associated with erectile dysfunction (ED) as an adverse effect and drugs used in the treatment of ED.

Radar plots were generated to visually compare physicochemical descriptors across the full panel of compounds. Chemical structures of selected representative drugs—clonazepam, fluoxetine, sildenafil, and diazepam—were drawn and energy-minimized using ChemDraw 3D (PerkinElmer, version 12.0, Waltham, MA, USA) to illustrate their structural diversity and relevance to their physicochemical behavior [21,22,23].

### 2.6. Statistical Analysis

Descriptive statistics were used to summarize participant characteristics. The normal distribution of the data was verified using the Kolmogorov–Smirnoff test. Continuous variables were expressed as means and standard deviations (SDs), while categorical variables were presented as frequencies and percentages. Comparisons between groups (with and without SDs) were performed using Student’s *t* test for continuous variables and Fisher’s exact test for categorical variables. Initially, crude odds ratios (ORs) with 95% confidence intervals (CIs) were calculated via bivariable logistic regression for each variable to explore unadjusted associations with erectile dysfunction (ED). Subsequently, a multivariable binary logistic regression with backward stepwise selection was carried out to identify the most parsimonious model, applying entry and removal p-thresholds of 0.05 and 0.10, respectively; only the final model is presented [24,25]. Spearman’s rank correlation coefficient (ρ) was used to assess the associations between the Insomnia Scale (AIS), depressive symptoms, and erectile dysfunction scores (IIEF-5) as the total scores constitute discrete numerical variables derived from ordinal-level items. To evaluate potential effect modification between insomnia and benzodiazepine use, the population were stratified into four mutually exclusive exposure groups: (1) no insomnia and no benzodiazepine use (reference group); (2) insomnia only; (3) benzodiazepine use only; and (4) both insomnia and benzodiazepine use. Variables included in the analyses were selected based on their established clinical relevance and consistent support in the scientific literature [26] regarding their association with ED, ensuring that important potential confounders were accounted for regardless of their statistical significance in crude analyses. Statistical analyses were performed using SPSS version 25 (IBM Corp., Armonk, NY, USA) [27], except for sample size, which was calculated using OpenEpi version 1 (https://www.openepi.com/SampleSize/SSCC.htm, accessed 15 April 2025) [28], and statistical power, which was calculated using ClinCalc version 1 (https://clincalc.com/stats/Power.aspx, accessed 18 January 2025). A *p* < 0.05 level was considered statistically significant [29]. No formal correction for multiple testing was applied due to the limited number of variables assessed and the focused, hypothesis-driven examination of key known predictors of ED [30,31]. This approach minimizes the risk of unnecessarily increasing Type II error and aligns with accepted epidemiological practices [30,31]. Therefore, when interpreting the statistical significance of the results, consideration should be given to the adjusted odds ratios—which represent the effect size—and their confidence intervals alongside *p*-values, to provide a comprehensive and clinically meaningful evaluation of the identified associations [32,33].

## 3. Results

### 3.1. Characteristics of the Study Population by Presence of Erectile Dysfunction

A total of 183 older adult men were included in the analysis (mean age 75.12 ± 7.24 years). Erectile dysfunction (ED) was present in 100 participants (54.6%) and absent in 83 (45.4%). Table 1 summarizes the demographic and clinical characteristics stratified by ED status. There were no significant differences in age (75.6 vs. 74.5 years; *p* = 0.305), proportion of participants aged ≥75 years (55.0% vs. 49.4%; *p* = 0.462), or prevalence of comorbidities such as diabetes, hypertension, smoking, or alcohol consumption between participants with and without ED.

However, insomnia (58.0% vs. 41.0%, *p* = 0.026) and benzodiazepine use (38.0% vs. 22.9%, *p* = 0.037) were significantly more frequent among those individuals with ED. In general, 30 patients (16.4%) were using antidepressants, all of which were selective serotonin reuptake inhibitors (SSRIs), primarily escitalopram (9.8%), followed by citalopram (3.3%) and sertraline (3.3%). The benzodiazepine clonazepam, which is primarily prescribed for insomnia and anxiety disorders, was used by 57 patients (31.1%). The presence of depression (five or more points on GDS at the time of evaluation) was higher in participants with ED (51.0% vs. 41.0%), the difference did not reach statistical significance (*p* = 0.184) (Table 1).

### 3.2. Independent Risk Factors for Erectile Dysfunction

In the multivariable logistic regression model, two variables remained significantly associated with ED: insomnia and benzodiazepine use. Both insomnia (aOR: 2.05; 95% CI: 1.13–3.74; *p* = 0.019) and benzodiazepine use (aOR: 2.14; 95% CI: 1.10–4.15; *p* = 0.025) were independently associated with an approximately twofold increase in the likelihood of ED. No other covariates, such as age >75 years, diabetes, hypertension, tobacco or alcohol use, or self-reported depression or antidepressant use, remained significant in the adjusted model (Table 2).

Spearman’s correlation analysis was conducted to assess the relationships between scores from the Athens Insomnia Scale (AIS) and the International Index of Erectile Function-5 (IIEF-5), noting that higher AIS scores indicate greater insomnia severity, while lower IIEF-5 scores reflect more severe erectile dysfunction.

A weak but statistically significant negative correlation was observed between insomnia and erectile dysfunction scores (r = −0.181, *p* = 0.014), indicating that higher levels of insomnia symptoms were associated with greater likelihood of reporting ED.

No significant correlations were found between the Geriatric Depression Scale (GDS) and either erectile dysfunction (r = −0.130, *p* = 0.080) or insomnia (r = −0.064, *p* = 0.393). These findings suggest that insomnia symptoms, as measured by the AIS, are more closely linked to ED scores. Although the correlation between depressive symptoms and ED approached statistical significance, it did not reach the conventional threshold in this study.

### 3.3. Stratified Analysis and Evaluation of Potential Effect Modification

To further assess whether the association between insomnia and erectile dysfunction (ED) varies according to benzodiazepine use, a stratified analysis was conducted (Table 3). Participants were grouped based on the presence or absence of insomnia and benzodiazepine use to evaluate their individual and combined effects on ED risk. Compared with the reference group (no insomnia and no benzodiazepine use), participants with insomnia alone had an increased risk of ED (aOR = 2.31; 95% CI: 1.13–4.72; *p* = 0.021), as did those using benzodiazepines alone (aOR = 2.59; 95% CI: 1.05–6.42; *p* = 0.040). The highest risk was observed in participants exposed to both factors (aOR = 3.96; 95% CI: 1.51–10.40; *p* = 0.005). The combined odds ratio for participants with both insomnia and benzodiazepine use was close to what would be expected based on the individual effects of each factor, indicating that their joint presence is associated with an increased risk of ED approximately in line with the sum of their separate associations.

### 3.4. Physicochemical Descriptor Analysis

A total of 25 physicochemical descriptors were calculated and analyzed across 17 pharmacological compounds, including benzodiazepines, antidepressants, beta-blockers, and phosphodiesterase-5 inhibitors. To illustrate the most relevant differences, Table 4 presents a focused comparison of key descriptor values across five representative drugs: clonazepam (used as the model benzodiazepine in this study), fluoxetine and propranolol (commonly associated with erectile dysfunction), and sildenafil and tadalafil (agents widely used to treat erectile dysfunction). These compounds were selected due to their clinical relevance and contrasting roles in the modulation of sexual function. Although fluoxetine was used as the representative SSRI for visual and comparative analysis, additional SSRIs—escitalopram, fluvoxamine, and paroxetine—were also included in the full descriptor dataset to enrich the analysis.

The analysis of calculated physicochemical descriptors revealed distinct patterns among the evaluated compounds. Sildenafil showed the highest molecular weight (MW = 476.59), topological polar surface area (TPSA = 115.29 Å^2^), and number of hydrogen bond acceptors (8), as well as the lowest predicted dermal penetration (log Kp = −8.36), reflecting its high polarity and peripheral activity. In contrast, propranolol had the lowest molecular weight (MW = 259.34) and one of the highest fractions Csp^3^ values (0.38), indicating a higher aliphatic character.

Among benzodiazepines, flurazepam had the highest molecular weight (MW = 387.88), number of rotatable bonds (6), and the highest predicted lipophilicity based on several logP models (e.g., Silicos-IT LogP = 5.2). Oxazepam and lorazepam, on the other hand, had relatively high TPSA values (61.69 Å^2^) and hydrogen bond donors (2), suggesting greater polarity and potential CNS activity [34].

Fluoxetine stood out for having the highest consensus logP (4.32), reflecting its high lipophilicity and affinity for CNS tissues. In terms of solubility, sildenafil and propranolol showed the best ESOL-predicted aqueous solubility (2.85 × 10^−1^ mg/mL and 1.24 × 10^−1^ mg/mL, respectively), while clonazepam and fluoxetine were among the least soluble (1.77 × 10^−2^ mg/mL and 1.34 × 10^−2^ mg/mL, respectively).

**Figure 1:**
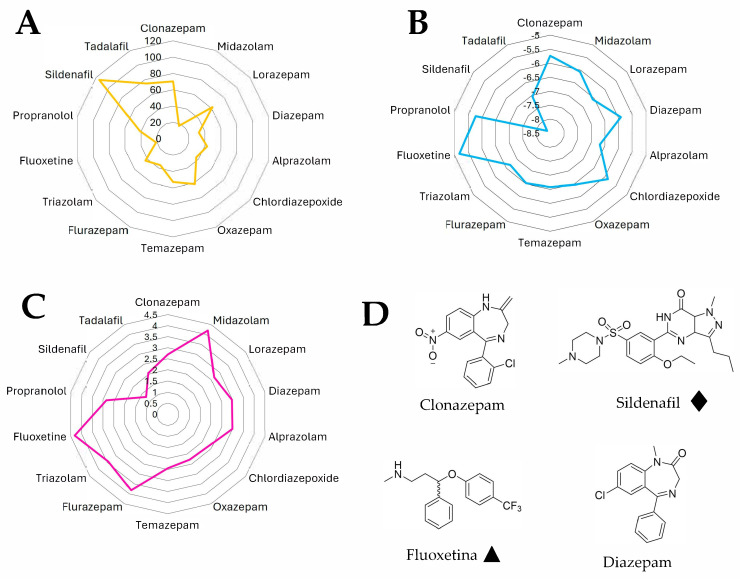
Physicochemical descriptor comparison among drugs with reported or potential influence on sexual function. (**A**) Topological polar surface area (TPSA). (**B**) Predicted skin permeability (log Kp). (**C**) Lipophilicity (consensus Log P) for a panel of 17 compounds including benzodiazepines, antidepressants, beta-blockers, and PDE5 inhibitors. (**D**) Representative chemical structures of selected compounds: clonazepam, fluoxetine, sildenafil, and diazepam, minimized using ChemDraw 3D. ♦ Drug recognized for therapeutic treatment of erectile dysfunction (ED). ▲ Drug associated with the onset or exacerbation of ED as a side effect.

Regarding skin permeability, tadalafil and sildenafil had the lowest log Kp values (−7.05 and −8.36, respectively), suggesting limited passive transdermal absorption, which contrasts with the higher log Kp values observed for fluoxetine (−5.18) and clonazepam (−5.74). This multidimensional analysis (Table 4 and Figure 1) highlights consistent physicochemical similarities between compounds associated with erectile dysfunction (e.g., fluoxetine, propranolol) [35,36], particularly in terms of high lipophilicity, moderate polarity, and favorable CNS permeability. In contrast, drugs used to treat erectile dysfunction (e.g., sildenafil, tadalafil) exhibit markedly different profiles, characterized by higher polarity, lower lipophilicity, and limited CNS penetration. These contrasting patterns may help explain their opposing pharmacological effects on erectile function.

## 4. Discussion

This analytical cross-sectional study in adults aged 65 years or older. We found that insomnia and benzodiazepine use were each independently associated with approximately a twofold increase in the odds of erectile dysfunction (ED), and their coexistence further amplified the risk (aOR 3.96), suggesting a possible synergistic effect of these two prevalent and potentially modifiable risk factors. In addition, although the correlation between depressive symptoms and erectile dysfunction did not reach statistical significance (*p* = 0.080), the result was near the threshold. This suggests a possible trend toward association that may not have reached significance due to sample size limitations. In contrast, the statistically significant correlation between insomnia severity and ED, supported by both bivariable and multivariable analyses, reinforces the central role of sleep disturbances—rather than depression per se—as a key psychological factor contributing to sexual dysfunction in this population.

Clonazepam, the predominant benzodiazepine used in this cohort, is frequently prescribed for insomnia and anxiety in older adults [12], and has also shown usefulness in cases of treatment-resistant depression [37]. Although less studied than serotonin reuptake inhibitors (SSRIs) [38] in terms of sexual side effects, it has been associated with decreased libido and erectile difficulties, likely through GABAergic [39] CNS depression [13].

SSRIs such as escitalopram, citalopram, and sertraline are well established to cause sexual dysfunction, including ED, via serotonergic enhancement that inhibits dopaminergic and noradrenergic neurotransmission critical for sexual arousal and performance [13].

The combined effect of insomnia and benzodiazepine use on ED risk observed in this study likely reflects complementary pathophysiological mechanisms. Insomnia disrupts the hypothalamic–pituitary–gonadal axis, leading to reduced testosterone levels and endothelial dysfunction mediated by oxidative stress, impairing vascular penile relaxation necessary for erection [40]. Concurrently, clonazepam may exacerbate sexual dysfunction primarily by enhancing GABA-A receptor function, which leads to a general central nervous system (CNS) depression and direct impairment of penile erection [12]. This CNS depression manifests as sedation, cognitive impairment, and reported dysfunctions including decreased libido, ED, delayed ejaculation, and anorgasmia. Notably, men undergoing such pharmacological interventions for insomnia were more frequently prescribed medication for ED, such as phosphodiesterase-5 inhibitors, highlighting the combined impact of these factors [4].

Age is a well-recognized independent risk factor for erectile dysfunction, with prevalence increasing significantly after 70 years of age (being present in up to 67% of men over 70 years of age) due to physiological changes such as vascular stiffness, decreased testosterone levels, and common comorbidities in older adults. However, since all participants in this study were aged 65 years or older [41], the effect of age as a risk factor was likely attenuated within this relatively homogenous older population. Therefore, while age remains an important consideration in the general context of ED, it did not emerge as a differentiating factor in the present analysis.

Clinically, these findings support the importance of evaluating sleep quality and medication use in older men with ED [42,43]. Non-pharmacological approaches such as cognitive–behavioral therapy for insomnia (CBT-I) may help address both sleep disturbances and associated sexual dysfunction [44]. When pharmacologic treatment is necessary, selecting agents with fewer sexual side effects or adjusting benzodiazepine use may reduce risk [42,43,44]. Additionally, careful selection of sleeping pills and anxiolytics with lower sexual side-effect profiles or dose adjustments may mitigate sexual dysfunction. For patients requiring benzodiazepines like clonazepam, minimizing dose and duration or considering alternative anxiolytics may reduce adverse sexual effects.

The inclusion of a physicochemical analysis in this study is justified by the growing need to understand how molecular properties influence drug distribution, particularly into the central nervous system (CNS), where many agents may interfere with neurovascular and hormonal pathways involved in erectile function. When analyzing the physicochemical descriptors of the drugs associated with erectile dysfunction, such as fluoxetine and propranolol, it becomes evident that they share notable similarities with clonazepam, a benzodiazepine also linked to ED. These compounds exhibit moderate to high lipophilicity (consensus logP ranging from 2.7 to 4.3), relatively low aqueous solubility, and low to moderate polar surface areas (TPSA < 80 Å^2^), favoring central nervous system (CNS) penetration and potential interference with neurovascular mechanisms involved in sexual function.

In contrast, sildenafil, a representative agent used to treat erectile dysfunction, displays markedly different characteristics: it has a high topological polar surface area (TPSA: 115.29 Å^2^), a greater number of hydrogen bond acceptors (8), and lower consensus logP (1.26), which reflects a lower lipophilicity and reduced CNS penetration. These physicochemical properties align with its targeted peripheral action on vascular smooth muscle and minimal central effects. Collectively, this comparison highlights that drugs inducing ED tend to cluster around physicochemical profiles resembling clonazepam, while agents like sildenafil exhibit distinctly opposing features that support their therapeutic efficacy in erectile function [34,35,36]. The inclusion of SSRIs such as paroxetine, fluoxetine, escitalopram, and fluvoxamine in the physicochemical analysis provided additional insights into how variations in lipophilicity and polar surface area may influence the risk of sexual dysfunction. Notably, paroxetine and fluvoxamine, which have relatively high polarity (TPSA) and lower membrane permeability, have been frequently associated with a higher incidence of sexual side effects. Conversely, escitalopram, although chemically similar, exhibited a slightly different physicochemical profile, which may partly explain reported differences in tolerability. These comparisons highlight that beyond their pharmacodynamic classifications, the physicochemical fingerprints of psychotropic drugs could serve as predictors for adverse sexual effects. Future studies incorporating broader compound libraries and in silico modeling could help identify high-risk molecules early in the drug development process or guide clinical decision-making in patients susceptible to ED.

This study has some limitations. First, its cross-sectional design limits the ability to draw causal inferences, and reverse causation cannot be ruled out—for example, erectile dysfunction (ED) itself may contribute to sleep disturbances or psychological distress, potentially increasing benzodiazepine use. Second, although multivariable models adjusted for key demographic and clinical variables, certain important potential confounders were not assessed, such as anxiety disorders, polypharmacy, cardiovascular disease severity, serum testosterone levels, antihypertensive medications, severe comorbidities, and socioeconomic status. These factors were mentioned only in the limitations section and were not operationally defined or measured in the present study, which constitutes a methodological limitation that may have influenced the observed associations. Finally, although the association between insomnia/benzodiazepine use and ED demonstrated good statistical power (83%), the relatively small sample size suggests that future studies with larger and more diverse cohorts are warranted to confirm these findings and account for a broader range of confounders.

## 5. Conclusions

In conclusion, this study highlights that insomnia and the use of commonly prescribed psychotropic medications—such as clonazepam—independently increase the risk of erectile dysfunction (ED), and that their co-occurrence is associated with an even greater risk in older men. These findings emphasize the importance of a multidisciplinary approach to managing sexual dysfunction in this population, incorporating comprehensive sleep assessment, careful pharmacological management, and patient education to optimize sexual health outcomes. Additionally, our in silico analysis of molecular descriptors provides mechanistic insight into how the physicochemical properties of these drugs may influence their ability to affect erectile function, supporting the clinical associations observed.

## Figures and Tables

**Table 1:** Clinical and demographic characteristics of participants stratified by presence of erectile dysfunction.

Variable	All	Erectile Dysfunction		*p*
		No (*n* = 83)	Yes (*n* = 100)	
Age (years)	75.12 + 7.24	74.51 + 7.09	75.62 + 7.36	0.305
≥75 years old	52.50%	49.40%	55.00%	0.462
Diabetes	51.4%	51.8%	51.0%	0.999
Hypertension	61.7%	65.1%	59.0%	0.447
Tobacco use	55.20%	56.60%	54.00%	0.766
Alcohol use	27.9%	27.7%	28.0%	0.999
Insomnia	50.3%	41.00%	58.00%	0.026
Depression	46.4%	41.00%	51.00%	0.184
Benzodiazepine use *	31.1%	22.9%	38.0%	0.037
Antidepressant (SSRIs) use **	16.4%	14.5%	18.0%	0.554

Data are presented as mean ± standard deviation or percentage, as appropriate. Comparisons between groups were performed using Student’s *t* test for continuous variables and Fisher’s exact test for categorical variables. * Clonazepam (a benzodiazepine). ** Selective serotonin reuptake inhibitors (SSRIs) antidepressant.

**Table 2:** Multivariable logistic regression of factors associated with erectile dysfunction.

	Bivariable Model				Multivariable Model			
	OR	95% CI		*p*	aOR	95% CI		*p*
		Lower	Upper			Lower	Upper	
>75 years old	1.25	0.70	2.24	0.450				
Type 2 Diabetes (DM2)	0.97	0.54	1.73	0.913				
Hypertension (HAS)	0.77	0.42	1.41	0.401				
Tobacco use	0.90	0.50	1.62	0.722				
Alcohol use	1.29	0.71	2.35	0.408				
Depression	1.50	0.83	2.70	0.176				
Insomnia	1.99	1.10	3.59	0.022	2.05	1.13	3.74	0.019
Benzodiazepine use *	2.06	1.08	3.96	0.029	2.14	1.10	4.15	0.025
Antidepressant (SSRIs) use **	1.29	0.58	2.88	0.520				

* Clonazepam (a benzodiazepine). ** Selective serotonin reuptake inhibitors (SSRIs) antidepressant. OR = odds ratio; aOR = adjusted odds ratio; CI = confidence interval; DM2 = type 2 diabetes mellitus; HAS = systemic arterial hypertension. In the multivariable model, only insomnia and benzodiazepine use remained statistically significant (*p* < 0.05).

**Table 3:** Stratified analysis of the combined effect of insomnia and benzodiazepine use on erectile dysfunction.

Group	*n*	Insomnia	Benzodiazepine Use	% with ED	aOR	95% CI		*p*
						Lower	Upper	
1 Reference	62	No	No	38.7%	Reference			
2 Insomnia only	64	Yes	No	62.1%	2.31	1.13	4.72	0.021
3 Benzodiazepine use only	29	No	Yes	59.4%	2.59	1.05	6.42	0.040
4 Combined effects	28	Yes	Yes	71.4%	3.96	1.51	10.40	0.005

aOR = adjusted odds ratio; CI = confidence interval. The reference group (no insomnia and no benzodiazepine use) served as the comparator. The joint effect of both exposures suggests an effect modification between these two factors.

**Table 4:** Comparative analysis of key physicochemical descriptors among selected drugs associated with erectile dysfunction and its treatment.

Parameter	Clonazepam	Associated with ED		Treatment of ED	
		Fluoxetine	Propranolol	Sildenafil	Tadalafil
MW	313.74	309.33	259.34	476.59	389.4
TPSA	70.21	21.26	41.49	115.29	74.87
LogP	2.7	4.32	2.84	1.26	2.05
# H-bond acceptors	3	5	3	8	4
# H-bond donors	1	1	2	1	1
# Rotatable bonds	2	7	6	7	1
Solubility	−4.25	−4.36	−3.32	−3.22	−4.01
log Kp	−5.74	−5.18	−5.77	−8.36	−7.05

MW: Molecular weight (g/mol); TPSA: topological polar surface area (Å^2^); LogP: predicted lipophilicity values; # H-bond acceptors: number of hydrogen bond acceptors; # H-bond donors: number of hydrogen bond donors; # Rotatable bonds: number of rotatable single bonds; Solubility: predicted solubility in mg/mL; log Kp: Predicted skin permeability (log cm/s).

## Data Availability

The original contributions presented in the study are included in the article; further inquiries can be directed to the corresponding author.
